# Oligogenic Inheritance of Monoallelic *TRIP11*, *FKBP10*, *NEK1*, *TBX5*, and *NBAS* Variants Leading to a Phenotype Similar to Odontochondrodysplasia

**DOI:** 10.3389/fgene.2021.680838

**Published:** 2021-06-02

**Authors:** Alice Costantini, Helena Valta, Anne-Maarit Suomi, Outi Mäkitie, Fulya Taylan

**Affiliations:** ^1^Department of Molecular Medicine and Surgery and Center for Molecular Medicine, Karolinska Institutet, Stockholm, Sweden; ^2^Children’s Hospital, Pediatric Research Center, University of Helsinki and Helsinki University Hospital, Helsinki, Finland; ^3^Department of Pediatrics, Seinäjoki Central Hospital, Seinäjoki, Finland; ^4^Department of Clinical Genetics, Karolinska University Hospital, Stockholm, Sweden; ^5^Folkhälsan Institute of Genetics, and Research Program for Clinical and Molecular Metabolism, University of Helsinki, Helsinki, Finland

**Keywords:** *TRIP11*, oligogenic inheritance, odontochondrodysplasia, achondrogenesis type IA, short stature

## Abstract

Skeletal dysplasias are often well characterized, and only a minority of the cases remain unsolved after a thorough analysis of pathogenic variants in over 400 genes that are presently known to cause monogenic skeletal diseases. Here, we describe an 11-year-old Finnish girl, born to unrelated healthy parents, who had severe short stature and a phenotype similar to odontochondrodysplasia (ODCD), a monogenic skeletal dysplasia caused by biallelic *TRIP11* variants. The family had previously lost a fetus due to severe skeletal dysplasia. Exome sequencing and bioinformatic analysis revealed an oligogenic inheritance of a heterozygous nonsense mutation in *TRIP11* and four likely pathogenic missense variants in *FKBP10*, *TBX5*, *NEK1*, and *NBAS* in the index patient. Interestingly, all these genes except *TBX5* are known to cause skeletal dysplasia in an autosomal recessive manner. In contrast, the fetus was found homozygous for the *TRIP11* mutation, and achondrogenesis type IA diagnosis was, thus, molecularly confirmed, indicating two different skeletal dysplasia forms in the family. To the best of our knowledge, this is the first report of an oligogenic inheritance model of a skeletal dysplasia in a Finnish family. Our findings may have implications for genetic counseling and for understanding the yet unsolved cases of rare skeletal dysplasias.

## Introduction

Skeletal dysplasias are genetically and phenotypically heterogenous diseases due to abnormal development of bone and cartilage. So far, the molecular etiology of over 92% of skeletal dysplasias has been explained by mutations in over 400 genes, and the conditions are mostly described as single-gene diseases (Mortier et al., 2019). However, a small group of genetically unsolved cases can be explained by mechanisms such as variable expressivity, incomplete or non-penetrance, methylation defects, somatic mosaicism, and di- or oligogenic inheritance models. Some of these mechanisms have been shown to cause skeletal dysplasia in a rather small number of families (Maupetit-Mehouas et al., 2010; Thiel et al., 2011; Mortier et al., 2019; Costantini et al., 2020), indicating that different genetic mechanisms can result in phenotypes that are similar to the ones explained by Mendelian inheritance models. Although oligogenic inheritance was commonly accepted to explain complex disorders, cumulative effect of rare variants in two or more genes acting in the same network and tissues can cause a phenotype similar to a monogenic disease (Kim et al., 2019).

Despite their phenotypic differences, achondrogenesis type IA (Smits et al., 2010; ACG1A; MIM 200600) and odontochondrodysplasia (Wehrle et al., 2019; ODCD; MIM 184260) are both caused by mutations of thyroid hormone receptor interactor 11 (*TRIP11*), which encodes the Golgi-associated microtubule-binding protein 210 (GMAP-210). GMAP-210 plays a pivotal role in maintaining the structure of the Golgi complex, in membrane tethering, and in vesicle trafficking (Smits et al., 2010; Sato et al., 2015). GMAP-210 is also involved in protein trafficking from the Golgi to the primary cilium by interacting with the intraflagellar transport 20 (IFT-20; Follit et al., 2008). While biallelic loss-of-function mutations of *TRIP11* lead to ACG1A (Smits et al., 2010), compound-heterozygous hypomorphic *TRIP11* mutations cause ODCD, a milder condition than ACG1A, which is usually perinatally lethal. Thus, ACG1A and ODCD are caused by a complete and a partial loss of GMAP-210 function, respectively.

In this report, we describe a Finnish family in which a fetus and the index child are affected by seemingly two different skeletal dysplasias. The phenotypes of the fetus and the index patient are explained by monogenic and oligogenic inheritance models after a detailed bioinformatic analysis of the exome sequencing data.

## Subjects and Methods

### Study Approval and Sample Collection

Our study was approved by the ethics committee of the Helsinki University Central Hospital and carried out according to the ethical principles of the Declaration of Helsinki (World Medical Association, 2013). The index patient was evaluated at the Children’s Hospital, Helsinki University Hospital for suspicion of skeletal dysplasia. Other available family members were also recruited. All subjects or their legal guardians gave an informed consent before participation in the study. Genomic DNA was extracted from the blood of the affected individual, healthy parents, and a sibling. Additionally, DNA was extracted from a formalin-fixed paraffin-embedded fetal tissue of the family’s deceased fetus.

### Methods

#### Exome Sequencing

To identify the genetic cause of skeletal dysplasia in this family, we carried out exome sequencing (ES). Libraries were prepared, and the data were processed as previously described (Taylan et al., 2016). Minor allele frequency (MAF) from genome aggregation database (gnomAD; Karczewski et al., 2020) and SweGen (Ameur et al., 2017) databases were used.

Variants were filtered for Mendelian inheritance models, MAF < 0.01 and impact severity equal to either high or medium in GEMINI (Paila et al., 2013). Additionally, a skeletal dysplasia panel (version 2.32) from Genomics England PanelApp (Martin et al., 2019) was used to filter variants in known skeletal dysplasia genes (Supplementary Table S1).

The fetal sample was only used for Sanger sequencing to investigate the presence of one candidate variant.

#### Bioinformatic Analysis for Oligogenic Inheritance and Gene Network Analysis

The Oligogenic Resource for Variant AnaLysis (ORVAL) platform (Renaux et al., 2019) was used, according to the developers’ recommendations, to predict the additive effect of rare variants affecting more than two genes and to provide evidence for oligogenic inheritance. Both heterozygous and homozygous rare variants in skeletal dysplasia genes were used as input (Supplementary Table S2), and data were analyzed in ORVAL according to the recommendations of the developers (Renaux et al., 2019).

Candidate variants suggesting oligogenic inheritance were further evaluated for pathogenicity using VarSome (Kopanos et al., 2019) and model organism aggregated resources for rare variant exploration (MARRVEL; Wang et al., 2017). Network analysis of the candidates was performed using GeneCodis 4.0 (Tabas-Madrid et al., 2012). Hypergeometric test and false discovery rate (FDR) < 0.05 were used to determine the enriched GO biological processes.

## Results

### Clinical Report

**Figure 1:**
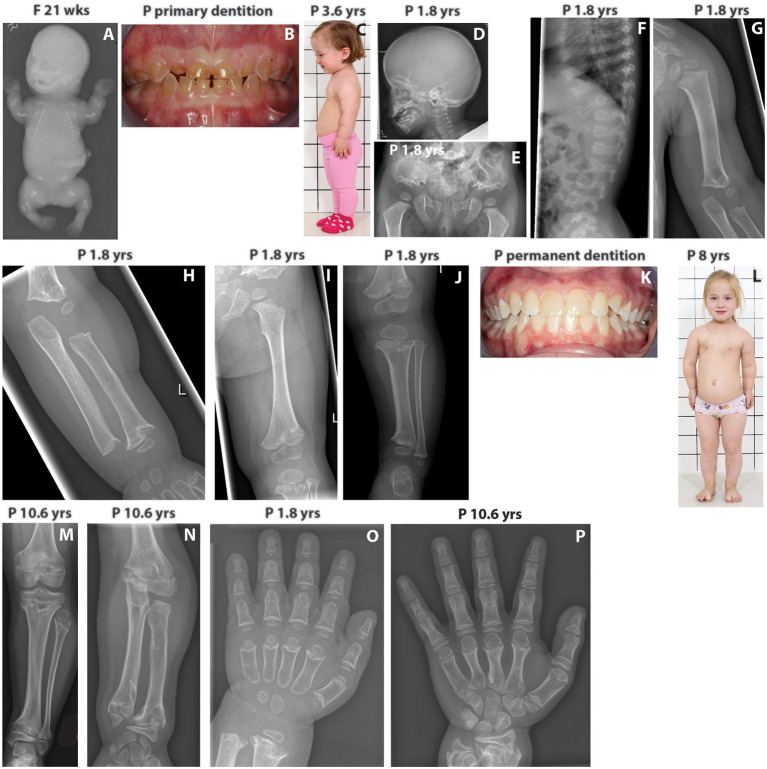
**(A)** Babygram of the deceased fetus shows extremely short tubular bones, very narrow thorax, horizontally oriented short ribs, and retarded ossification of the vertebrae. **(B)** Patient’s primary dentition showing dentinogenesis imperfecta. **(C)** The index patient at 3.6 yrs has short neck and limbs, redundant skin folds in the arms, and protuberant abdomen. **(D)** The skull of the index shows mild frontal bossing. **(E)** Patient’s pelvis showing trident configuration of the acetubulum and lacy iliac wings as well as short femoral necks with metaphyseal irregularities. **(F)** Patient’s spine displaying abnormal vertebrae with coronal clefts. **(G–I)** X-rays showing short and broad humerus with metaphyseal changes and shortening of the tibia and ulna as well as flared metaphyses. **(J)** The lower limb of the index patient displays short long bones, short femoral neck, marked metaphyseal irregularities, and normal epiphyses. **(K)** Patient’s permanent dentition with no signs of dentinogenesis imperfecta. **(L)** The index at the age of 8 yrs has normal facial features, short neck, redundant skin folds in the arms, narrow chest, prominent sternum, short limbs, mesomelic shortening of the upper arm and brachydactyly. **(M)** At 10.6 yrs, the index patient has marked metaphyseal irregularities of the tibia and ulna. **(N)** At 10.6 yrs, overgrowth of the fibula is noticed. **(O,P)** Hands of the index at 1.8 and 10.6 yrs. Brachydactyly and distinctive changes with deeper cupping of the metaphyses are evident. Progressive metaphyseal changes of the distal radius and ulna are also seen at 10.6 yrs. F, fetus; P, index patient; wks, weeks; yrs, years.

The index girl is the first child born to healthy non-consanguineous Finnish parents who also have a healthy son. The couple’s first pregnancy was terminated at 21 weeks of gestation due to fetal ultrasounds showing extremely short tubular bones and narrow thorax; postmortem radiological findings were consistent with ACG1A (Figure 1A).

In the index patient, fetal ultrasounds detected short tubular bones, but head and thorax circumferences were normal. The pregnancy and delivery at 40 weeks of gestation were otherwise uneventful, and birth measurements were normal: weight 3,570 g (+0.1 SD), length 47 cm (−1.8 SD), and head circumference 36.9 cm (+0.9 SD). She had a narrow thorax and short extremities, but skeletal features were not consistent with ACG1A. Abdominal, heart, and brain ultrasounds were normal. Genetic testing for *FGFR3*, *RMRP*, and *COL2A1* mutations was negative.

She developed progressive short stature, and at the age of 1.8 years, her length was 70.3 cm (−5.4 SD) and head circumference 49 cm (+0.6 SD). Her forehead was mildly prominent, but facial features were normal. The primary teeth were translucent and brownish consistent with dentinogenesis imperfecta (Figure 1B). During infancy, the limbs showed rhizomelic shortening, the thorax was narrow, and the ribs short (Figure 1C). She had joint laxity and mild tibial bowing. Neurological, gross, and fine motor skills were normal. Radiographs revealed mild frontal bossing, trident configuration of the pelvis, lacy iliac wings, abnormal vertebrae with coronal clefts and severe metaphyseal abnormalities with cupping in the tubular bones (Figures 1D–J). Her spondylometaphyseal dysplasia with dentinogenesis imperfecta suggested the diagnosis of odontochondrodysplasia.

As a toddler, she had recurrent middle ear infections but no pneumonia or episodes of wheezing. The oscillometry was normal. Laboratory tests including full blood count, immunoglobulins, plasma phosphate, alkaline phosphatase, calcium, and serum 25-hydroxy vitamin D as well as kidney and liver function were all normal.

Her permanent dentition had no signs of dentinogenesis imperfecta (Figure 1K), but she developed mesomelic shortening of the limbs and mild pectus carinatum (Figure 1L). Her thorax was narrow and short. Her neck was short and broad, but there was no instability of the cervical spine. Her thorax was narrow and short, her long bones featured metaphyseal irregularities (Figures 1M,N). Overgrowth of the fibula was also noticed (Figure 1N). Due to progressive lumbar kyphosis and scoliosis, brace treatment was started at 11 years. However, scoliosis progressed (Cobb angle of 44 degrees), and a spinal surgery has been scheduled. Proneness to respiratory infections alleviated at school age. Neuro-ophthalmological screenings and hearing have been normal.

At 11.7 years, she is in early puberty (Tanner M2), and her height is 114.7 cm (−5.3 SD). She has brachydactyly (Figures 1O,P), while her hair, eyelashes, and nails are normal. Her cognitive development has been normal with very good school performance.

### Genetic Findings

**Figure 2:**
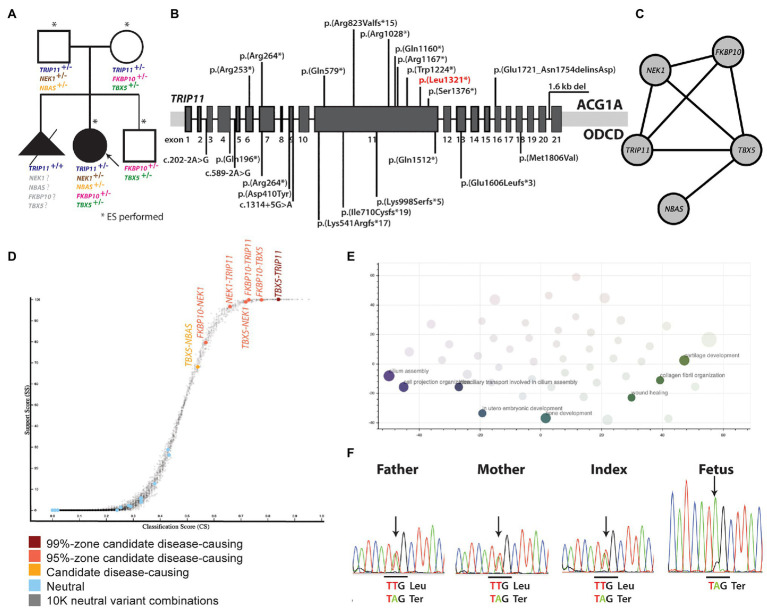
**(A)** Pedigree of the family and rare mutations identified in the genes presently linked to skeletal dysplasia. **(B)** Biallelic *TRIP11* mutations that are presently known to cause ACG1A and ODCD. The mutation that has also been identified in the present study is marked with red. **(C)** Gene network showing the interaction between the genes in which rare mutations have been identified. **(D)** Genes affected by variants suggesting digenic inheritance. **(E)** Biological processes in which the genes suggesting oligogenic inheritance are involved. **(F)** Sanger sequencing of the *TRIP11* region affected by the nonsense p.(Leu1321*) mutation. ACG1A, achondrogenesis type IA; ODCD, odontochondrodysplasia.

Exome sequencing included the index patient, parents, and the healthy sibling (Figure 2A). Extensive analysis of the exome for Mendelian inheritance models identified rare variants fitting to autosomal recessive pattern with unknown significance in five genes not yet linked to skeletal dysplasia (Supplementary Table S3). Besides, one *de novo* variant of unknown significance was identified. Comprehensive evaluation of existing data on the function and expression pattern of each gene did not provide evidence for a connection between gene defects and patient’s phenotype.

Eighty-seven rare variants in skeletal dysplasia genes were identified (Supplementary Table S2), and seven of them (Supplementary Table S4) were classified as high or medium impact variant by Variant Effect Predictor (McLaren et al., 2016). However, none of them alone could explain the phenotype because no single variant in a gene was fitting into the expected inheritance model for the associated disease.

Based on suspicion of odontochondrodysplasia in the index patient and of achondrogenesis type 1A in the fetus, *TRIP11* was an obvious candidate gene. However, ES data indicated the presence of a monoallelic nonsense mutation, p(Leu1321*), in the index patient (Figure 2A), which was previously reported (Wehrle et al., 2019; Figure 2B). As the initial analysis failed to identify a single causative gene for the patient’s phenotype, we explored the potential digenic or oligogenic pathogenic effects of the 87 rare variants. Interestingly, five monoallelic variants in *TRIP11*, *TBX5*, *NEK1*, *FKBP10*, and *NBAS* showed high support scores (>65) in ORVAL (Figure 2C). Moreover, different digenic combinations of these genes predicted as disease causing with a high confidence (Figure 2D; Supplementary Table S5). As presented in Table 1, all the variants involved highly conserved amino acids and were predicted as deleterious or disease causing by many *in silico* prediction tools. The nonsense *TRIP11* variant was either maternally or paternally inherited, as both parents were heterozygous for the same variant (Figure 2A). While *FKBP10* and *TBX5* variants were maternally inherited, *NEK1* and *NBAS* variants were paternally inherited. The healthy brother inherited only the maternal *FKBP10* and *TBX5* variants. The missense variant in *FKBP10* also affects the splice donor site in exon 4; thus, it is likely to lead to a splicing defect. As the parents and the brother are healthy, digenic combinations of these variants were excluded as having a pathogenic effect. Since a unique combination of these five variants exclusively exists in the patient, oligogenic inheritance could be the sole plausible model that could explain the peculiar phenotype of the patient (Figure 2C).

**Table 1:** Overview of the rare variants in skeletal dysplasia genes predicted to fit into oligogenic inheritance in the index patient.

	Rare variants				
Chromosome	17	14	2	4	12
Coordinate	39,976,713	92,470,358	15,470,841	170,520,286	114,837,349
Reference	C	A	T	T	C
Alternative	T	T	C	C	A
dbSNP id	rs146422412	rs745372938	rs143724414	rs201350526	rs77357563
Gene	*FKBP10*	*TRIP11*	*NBAS*	*NEK1*	*TBX5*
OMIM Phenotype	Bruck syndrome 1 (MIM 259450) Osteogenesis imperfecta, type XI (MIM 610968)	Achondrogenesis type 1A (MIM 200600) Osteochondrodysplasia (MIM 184260)	Infantile liver failure syndrome 2 (MIM 616483) Short stature, optic nerve atrophy, and Pelger-Huet anomaly (MIM 614800)	Short-rib thoracic dysplasia 6 with or without polydactyly (MIM 263520)	Holt-Oram syndrome (MIM 142900)
Variant at mRNA level	NM_021939.4:c.1256C > T	NM_004239.4:c.3962 T > A	NM_015909.4:c.4228A > G	NM_001199397.3:c.277A > G	NM_181486.4:c.331G > T
Variant at amino acid level	p.(Ser419Leu)	p.(Leu1321Ter)	p.(Thr1410Ala)	p.(Asn93Asp)	p.(Asp111Tyr)
Impact	Missense, near splice site	Stop-gain	Missense	Missense	Missense
Impact severity	Medium	High	Medium	Medium	Medium
ACMG Classification	Likely pathogenic	Pathogenic	Uncertain significance	Uncertain significance	Benign
PolyPhen	Probably damaging	NA	Possibly damaging	Probably damaging	Probably damaging
SIFT	Deleterious	NA	Deleterious	Deleterious	Deleterious
EIGEN	Pathogenic	Benign	Pathogenic	Pathogenic	Pathogenic
FATHMM-MKL	Damaging	Neutral	Damaging	Damaging	Damaging
Mutation Taster	Disease causing	Disease causing	Disease causing	Disease causing	Disease causing
BayesDel noAF	Damaging	Damaging	Tolerated	Tolerated	Damaging
PROVEAN	Damaging	NA	Damaging	Damaging	Damaging
M-CAP	Damaging	NA	Damaging	Damaging	Damaging
CADD v1.4	35	35	23.4	24.6	25.9
DANN	0.9993	0.9676	0.9975	0.9981	0.9953
dbNSFP rank score	0.754	0.314	0.562	0.738	0.676
REVEL	0.539	NA	0.108	0.455	0.874
GERP	4.889999866	−1.470000029	4.449999809	5.909999847	4.739999771
MAF gnomad all	0.00110887	0.000146471	0.00144686	0.000618097	0.00337435
MAF gnomad Finnish	0.00131293	0.001526032	0.000852018	0.006269168	0.000852018
Heterozygous	Mother, index, brother	Father, mother, index, brother	Father, index	Father, index	Mother, index, brother

OMIM, Online Mendelian inheritance in man; ACGM, American College of Medical Genetics; SIFT, sorting intolerant from tolerant; PROVEAN, protein variation effect analyzer; M-CAP, Mendelian clinically applicable pathogenicity; CADD, combined annotation dependent depletion; dbNSFP, database non-synonymous single-nucleotide variants; REVEL, rare exome variant ensemble learner; GERP, genomic evolutionary rate profiling; MAF, minor allele frequency; and gnomAD, genome aggregation database.

Enrichment analysis of the oligogenic combination network for biological processes showed the enrichment for cilium assembly, bone and cartilage development, cell projection organization, collagen fibril organization, *in utero* embryonic development, and chondrocyte differentiation involved in endochondral bone morphogenesis (Figure 2E; Supplementary Table S6).

As both parents were heterozygous carriers for the nonsense *TRIP11* mutation, which has already been linked to ACG1A (Wehrle et al., 2019), the fetus was independently investigated by Sanger sequencing for the presence of this mutation. Not surprisingly, the fetus was homozygous for the *TRIP11* mutation and the ACG1A diagnosis, which was originally suspected on the radiological findings, and was finally confirmed at molecular level (Figure 2F).

## Discussion

Although skeletal dysplasias are both genetically and phenotypically heterogeneous (Mortier et al., 2019), so far, the majority are described as monogenic. However, complex biological pathways participate in the development and maintenance of bone and cartilage tissue. Therefore, any minor disturbance caused by more than one gene can collectively have a noticeable impact on the skeletal phenotype.

Here, we describe five monoallelic variants affecting important pathways involved in bone development in an 11-year-old girl with a phenotype characterized by short stature, dentinogenesis imperfecta in the primary dentition, narrow thorax, short ribs, scoliosis, and mesomelic shortening of the limbs. The phenotype resembles ODCD, which is caused by biallelic *TRIP11* mutations. Since the patient only harbored a heterozygous *TRIP11* mutation, we searched for other potential gene variants contributing to her skeletal dysplasia.

Some of the patient’s clinical features overlap with diseases caused by mutations in *FKBP10*, *NEK1*, *TBX5*, and *NBAS* (Supplementary Table S7). Biallelic mutations of *FKBP10* cause osteogenesis imperfecta (MIM 610968; Alanay et al., 2010) or Bruck syndrome (MIM 259450; Shaheen et al., 2010), two allelic conditions with bone fragility and skeletal impairments. *FKBP10* encodes the 65-kDa FK506-binding protein, which acts as a molecular chaperon of type I collagen, the most abundant protein within the bone extracellular matrix (Barnes et al., 2012). Monoallelic variants of the T-Box transcription factor 5 (*TBX5*) are responsible for the Holt-Oram syndrome (MIM 142900), which is characterized by skeletal impairments of the upper limbs as well as congenital heart defects (Li et al., 1997). Since the mother and brother are healthy, and the patient does not have any heart-related problems, the *TBX5* variant alone was excluded as being disease causing for the Holt-Oram syndrome in this family. However, this variant could act as a genetic modifier by altering the gene expression of the other genes as it has been shown in other families with diseases caused by oligogenic inheritance (Gifford et al., 2019). Biallelic variants in both *NEK1* and *DYNC2H1* cause short-rib thoracic dysplasia (MIM 263520 and 613091; Dagoneau et al., 2009; Thiel et al., 2011), a disease that resembles the one observed in the index patient. *NEK1* encodes the serine/threonine-protein kinase Nek1, and it plays a pivotal role in DNA damage repair, in regulating the cell cycle, as well as in the formation of the primary cilium (Shalom et al., 2008; Chen et al., 2011). Interestingly, digenic inheritance of a heterozygous *NEK1* variant and a heterozygous variant in the cilia gene encoding a large dynein protein (*DYNC2H1*) has been reported in a patient with short-rib thoracic dysplasia (Thiel et al., 2011). Biallelic variants of *NBAS*, encoding the neuroblastoma-amplified gene protein, are linked to SOPH syndrome (MIM 614800), characterized by short stature, optic nerve atrophy, and Pelger-Huet anomaly (Maksimova et al., 2010). This gene is involved in the Golgi-to-endoplasmic reticulum (ER) retrograde transport (Aoki et al., 2009). None of the variants alone is sufficient to explain the phenotype of the patient, and the described OMIM phenotypes are not the same as the one seen in the patient. However, the synergistic cumulative effect of these variants could lead to the patient’s skeletal phenotype. In particular, the missense variant identified in *FKBP10* has a high combined annotation dependent depletion (CADD) score, and it is classified as likely pathogenic according to the American College of Medical Genetics (ACGM) guidelines. Thus, it could be a major contributor to the disease together with the pathogenic variant in *TRIP11*.

The unique combination of the monoallelic pathogenic variants in these genes could play an important role in determining short stature and causing ODCD through disturbing essential pathways in bone and cartilage development, cilium assembly, collagen fibril organization, among many others. Interestingly, the function of GMAP-210 partly overlaps with the function of NEK1 and NBAS. Both GMAP-210 and NEK1 play a role in the maintenance and assembly of the primary cilium, and both GMAP-210 and NBAS are involved in the protein trafficking to and from the Golgi. To the best of our knowledge, this is the first family where oligogenic inheritance model can explain the skeletal phenotype of a patient with a phenotype resembling ODCD.

Finally, we confirm the diagnosis of ACG1A in the fetus, who inherited the same pathogenic nonsense variant in *TRIP11* from the healthy parents. Since the parents harbor the same *TRIP11* variant despite not being related, it is likely that they share a common ancestor. This phenomenon is very common among Finnish families due to recent bottleneck effects. Finland is, in fact, an isolated population, and several genetic variants that are rare among the general population have a higher minor allele frequency in the Finnish population (Norio, 2003).

In conclusion, by using an oligogenic inheritance approach to analyze the ES data, we identified pathogenic variants in five skeletal dysplasia genes in a patient with a phenotype resembling ODCD and additionally found the genetic cause of disease in a fetus with ACG1A in the same family. The skeletal dysplasia in the index patient is likely due to a cumulative effect of pathogenic variants in multiple genes playing a pivotal role in bone development. Our findings may have valuable contributions to the field of bone diseases, implications in genetic counseling, and in understanding the still unsolved cases of rare skeletal dysplasias.

## Data Availability Statement

Most of the data relevant to the study are included in the article or uploaded as Supplementary Material. A signed informed consent has been obtained by the parents of the patient for inclusion of the clinical data. The data that support the findings of this study are available upon a reasonable request from the corresponding author.

## Ethics Statement

The studies involving human participants were reviewed and approved by Ethics Committee of the Helsinki University Central Hospital. Written informed consent to participate in this study was provided by the participants’ legal guardian/next of kin. Written informed consent was obtained from the minor(s)’ legal guardian/next of kin for the publication of any potentially identifiable images or data included in this article.

## Author Contributions

OM and FT designed the study. HV, A-MS, and OM collected and interpretated the clinical data of the patients. AC, FT, and OM conducted the study and performed the data analysis. AC, HV, OM, and FT interpreted the data and drafted the manuscript. All authors contributed to the article and approved the submitted version.

### Conflict of Interest

The authors declare that the research was conducted in the absence of any commercial or financial relationships that could be construed as a potential conflict of interest.
