# Supplementary material for: Oligogenic Inheritance of Monoallelic TRIP11, FKBP10, NEK1, TBX5, and NBAS Variants Leading to a Phenotype Similar to Odontochondrodysplasia
Source: Front Genet. 2021 Jun 2;12:680838. doi: 10.3389/fgene.2021.680838 (PMC8206634; doi:10.3389/fgene.2021.680838)
Supplement: Supplementary file 1 [file Data_Sheet_1.DOCX]

**Supplemental material**

**Oligogenic inheritance of monoallelic *TRIP11*, *FKBP10*, *NEK1*, *TBX5* and *NBAS* variants leading to a phenotype similar to odontochondrodysplasia**

Costantini et al**.**

**Supplemental Tables**

**Supplemental Table S1.** Genes targeted by the Skeletal Dysplasia Gene Panel (green panel, version 2.32) from Genomics England PanelApp.

| *ABCC9* | *CASR* | *DSPP* | *GNPTAB* | *MAN2B1* | *PHGDH* | *SH3PXD2B* | *TNFRSF11B* |
| --- | --- | --- | --- | --- | --- | --- | --- |
| *ACAN* | *CC2D2A* | *DVL1* | *GNPTG* | *MAP3K7* | *PIGT* | *SHOX* | *TNFSF11* |
| *ACP5* | *CCDC8* | *DVL3* | *GNS* | *MASP1* | *PIGV* | *SKI* | *TP63* |
| *ACVR1* | *CDC45* | *DYM* | *GORAB* | *MATN3* | *PIK3C2A* | *SLC10A7* | *TRAPPC2* |
| *ADAMTS10* | *CDH3* | *DYNC2H1* | *GPC6* | *MEGF8* | *PIK3R1* | *SLC17A5* | *TREM2* |
| *ADAMTS17* | *CDKN1C* | *DYNC2LI1* | *GSC* | *MEOX1* | *PITX1* | *SLC26A2* | *TRIP11* |
| *ADAMTSL2* | *CDT1* | *EBP* | *GUSB* | *MESP2* | *PLOD2* | *SLC29A3* | *TRPS1* |
| *AGA* | *CEP120* | *EED* | *HDAC8* | *MGP* | *PLS3* | *SLC34A1* | *TRPV4* |
| *AGPS* | *CEP290* | *EFTUD2* | *HES7* | *MKKS* | *POC1A* | *SLC34A3* | *TRPV6* |
| *ALG12* | *CHST14* | *EIF2AK3* | *HGSNAT* | *MKS1* | *POLR1A* | *SLC35C1* | *TTC21B* |
| *ALG3* | *CHST3* | *ENPP1* | *HOXA13* | *MMP13* | *POLR1C* | *SLC35D1* | *TTC8* |
| *ALG9* | *CHSY1* | *EOGT* | *HOXD13* | *MMP2* | *POLR1D* | *SLC39A13* | *TWIST1* |
| *ALPL* | *CLCN5* | *ERF* | *HPGD* | *MNX1* | *POP1* | *SLCO2A1* | *TYROBP* |
| *ALX1* | *CLCN7* | *ESCO2* | *HSPG2* | *MPDU1* | *POR* | *SMAD3* | *VDR* |
| *ALX3* | *COG1* | *EVC* | *ICK* | *MSX2* | *PPIB* | *SMAD4* | *WDPCP* |
| *ALX4* | *COL10A1* | *EVC2* | *IDH1* | *MYCN* | *PRKAR1A* | *SMARCAL1* | *WDR19* |
| *AMER1* | *COL11A1* | *EXT1* | *IDS* | *NAGLU* | *PRMT7* | *SMC1A* | *WDR34* |
| *ANKH* | *COL11A2* | *EXT2* | *IDUA* | *NANS* | *PSAT1* | *SMC3* | *WDR35* |
| *ANKRD11* | *COL1A1* | *EXTL3* | *IFIH1* | *NBAS* | *PSPH* | *SMOC1* | *WDR60* |
| *ANO5* | *COL1A2* | *EZH2* | *IFITM5* | *NEK1* | *PTDSS1* | *SNRPB* | *WISP3* |
| *ANTXR2* | *COL2A1* | *FAM111A* | *IFT122* | *NEU1* | *PTH1R* | *SNX10* | *WNT1* |
| *ARHGAP31* | *COL9A1* | *FAM20C* | *IFT140* | *NF1* | *PTHLH* | *SOST* | *WNT10B* |
| *ARL6* | *COL9A2* | *FAM46A* | *IFT172* | *NFIX* | *PTPN11* | *SOX9* | *WNT5A* |
| *ARSB* | *COL9A3* | *FAM58A* | *IFT43* | *NIPBL* | *PUF60* | *SP7* | *WNT7A* |
| *ARSE* | *COLEC11* | *FBN1* | *IFT52* | *NKX3-2* | *PYCR1* | *SPARC* | *XRCC4* |
| *ASXL1* | *COMP* | *FBN2* | *IFT80* | *NLRP3* | *RAB23* | *SUMF1* | *XYLT1* |
| *ASXL2* | *CREB3L1* | *FERMT3* | *IFT81* | *NOG* | *RAB33B* | *TALDO1* | *XYLT2* |
| *ATP6V0A2* | *CREBBP* | *FGF10* | *IHH* | *NOTCH1* | *RASGRP2* | *TAPT1* | *YY1* |
| *ATP7A* | *CRTAP* | *FGF16* | *IKBKG* | *NOTCH2* | *RBM8A* | *TBCE* | *ZMPSTE24* |
| *B3GALT6* | *CSPP1* | *FGF23* | *IL11RA* | *NPR* | *RBPJ* | *TBX15* | *ZSWIM6* |
| *B3GAT3* | *CTSA* | *FGFR1* | *IL1RN* | *NSD1* | *RECQL4* | *TBX3* |  |
| *B3GLCT* | *CTSC* | *FGFR2* | *IMPAD1* | *NSDHL* | *RFT1* | *TBX4* |  |
| *B4GALT7* | *CTSK* | *FGFR3* | *INPPL1* | *OBSL1* | *RMRP* | *TBX5* |  |
| *BBS1* | *CUL7* | *FIG4* | *KAT6B* | *OFD1* | *RNU4ATAC* | *TBX6* |  |
| *BBS10* | *CYP27B1* | *FKBP10* | *KIAA0753* | *ORC1* | *ROR2* | *TBXAS1* |  |
| *BBS12* | *CYP2R1* | *FLNA* | *KIF22* | *ORC4* | *RPGRIP1L* | *TCIRG1* |  |
| *BBS2* | *DDR2* | *FLNB* | *KIF7* | *ORC6* | *RPL13* | *TCOF1* |  |
| *BBS4* | *DHCR24* | *FN1* | *KMT2D* | *OSTM1* | *RUNX2* | *TCTEX1D2* |  |
| *BBS5* | *DHCR7* | *FUCA1* | *LBR* | *P3H1* | *SALL1* | *TCTN2* |  |
| *BBS7* | *DHODH* | *FZD2* | *LEMD3* | *P4HB* | *SALL4* | *TCTN3* |  |
| *BBS9* | *DIS3L2* | *GALNS* | *LIFR* | *PAPSS2* | *SBDS* | *TERT* |  |
| *BHLHA9* | *DLL3* | *GALNT3* | *LMBR1* | *PAX3* | *SCARF2* | *TGFB1* |  |
| *BMP1* | *DLL4* | *GDF5* | *LMNA* | *PCNT* | *SEC24D* | *TGFB2* |  |
| *BMP2* | *DLX3* | *GDF6* | *LMX1B* | *PCYT1A* | *SERPINF1* | *TGFBR2* |  |
| *BMPER* | *DLX5* | *GHR* | *LONP1* | *PDE3A* | *SERPINH1* | *TMCO1* |  |
| *BMPR1B* | *DMP1* | *GJA1* | *LPIN2* | *PDE4D* | *SETD2* | *TMEM165* |  |
| *C21orf2* | *DNMT3A* | *GLB1* | *LRP4* | *PEX5* | *SF3B4* | *TMEM216* |  |
| *C2CD3* | *DOCK6* | *GLI3* | *LRP5* | *PEX7* | *SFRP4* | *TMEM231* |  |
| *CA2* | *DPAGT1* | *GNAS* | *LTBP3* | *PGM3* | *SGSH* | *TMEM38B* |  |
| *CANT1* | *DPM1* | *GNPAT* | *MAFB* | *PHEX* | *SH3BP2* | *TNFRSF11A* |  |

**Supplemental Table S2.** Rare variants (n=87) identified in skeletal dysplasia genes that were used as input for ORVAL

| Chromosome | Position | Reference | Alternative | Zygosity |
| --- | --- | --- | --- | --- |
| 1 | 1275291 | G | A | Heterozygous |
| 1 | 103380383 | A | AG | Heterozygous |
| 1 | 103435626 | ATG | A | Heterozygous |
| 1 | 120277576 | G | A | Heterozygous |
| 1 | 120612154 | C | G | Heterozygous |
| 1 | 120612163 | C | A | Homozygous |
| 1 | 247582464 | TATA | T | Homozygous |
| 2 | 15470841 | T | C | Heterozygous |
| 2 | 15491994 | G | GCACA | Homozygous |
| 2 | 20126666 | ACC | A | Homozygous |
| 2 | 20193922 | A | G | Heterozygous |
| 2 | 170356145 | A | G | Heterozygous |
| 2 | 176959113 | G | GCA | Heterozygous |
| 3 | 30732989 | G | A | Heterozygous |
| 3 | 46935296 | G | A | Heterozygous |
| 3 | 57994597 | C | G | Heterozygous |
| 3 | 58090900 | G | A | Heterozygous |
| 3 | 145790064 | CAT | C | Homozygous |
| 3 | 189507518 | C | CAG | Heterozygous |
| 3 | 189507518 | C | CAGAG | Heterozygous |
| 3 | 195954077 | T | TC | Heterozygous |
| 3 | 195954080 | G | T | Heterozygous |
| 3 | 195956728 | AAG | A | Heterozygous |
| 4 | 15602771 | C | CTT | Heterozygous |
| 4 | 15602773 | AC | A | Heterozygous |
| 4 | 39187522 | TG | T | Heterozygous |
| 4 | 39187522 | G | T | Heterozygous |
| 4 | 39257671 | T | C | Heterozygous |
| 4 | 88532184 | AGT | A | Heterozygous |
| 4 | 88535073 | T | C | Heterozygous |
| 4 | 170428334 | G | GT | Heterozygous |
| 4 | 170428352 | TG | T | Heterozygous |
| 4 | 170458752 | C | T | Homozygous |
| 4 | 170483144 | GT | G | Homozygous |
| 4 | 170520286 | T | C | Heterozygous |
| 5 | 42467882 | C | A | Heterozygous |
| 5 | 60827204 | T | C | Heterozygous |
| 5 | 60831581 | T | G | Homozygous |
| 5 | 67575325 | ATT | A | Heterozygous |
| 5 | 67584512 | A | AT | Heterozygous |
| 5 | 122727171 | G | T | Homozygous |
| 5 | 127622609 | G | GT | Heterozygous |
| 5 | 127653974 | C | G | Heterozygous |
| 5 | 174155998 | G | GTA | Homozygous |
| 5 | 176715649 | T | C | Heterozygous |
| 5 | 177031602 | C | G | Homozygous |
| 6 | 74310232 | CT | C | Heterozygous |
| 6 | 137143709 | T | C | Homozygous |
| 7 | 96649884 | A | AG | Homozygous |
| 8 | 116504686 | CTG | C | Homozygous |
| 8 | 116632411 | GAA | G | Homozygous |
| 9 | 108472972 | A | G | Homozygous |
| 11 | 17131918 | T | G | Heterozygous |
| 11 | 22301412 | CTT | C | Homozygous |
| 11 | 44151408 | G | T | Heterozygous |
| 11 | 71146999 | G | A | Heterozygous |
| 12 | 4488369 | GCA | G | Homozygous |
| 12 | 48375664 | C | T | Heterozygous |
| 12 | 48376447 | A | G | Heterozygous |
| 12 | 48380331 | T | TG | Heterozygous |
| 12 | 48380684 | G | T | Heterozygous |
| 12 | 48383790 | CT | C | Heterozygous |
| 12 | 88519236 | G | C | Heterozygous |
| 12 | 88530707 | CT | C | Heterozygous |
| 12 | 114837349 | C | A | Heterozygous |
| 12 | 115112760 | G | A | Heterozygous |
| 14 | 76488655 | T | C | Heterozygous |
| 14 | 92470358 | A | T | Heterozygous |
| 15 | 100693033 | CT | C | Heterozygous |
| 16 | 29809638 | TACAC | T | Homozygous |
| 16 | 68371179 | AT | A | Heterozygous |
| 16 | 68380892 | TAGG | T | Heterozygous |
| 16 | 88907498 | G | A | Heterozygous |
| 16 | 89389872 | G | C | Heterozygous |
| 17 | 39975209 | CAA | C | Homozygous |
| 17 | 39975234 | T | G | Heterozygous |
| 17 | 39976713 | C | T | Heterozygous |
| 17 | 76993393 | C | T | Heterozygous |
| 18 | 2931063 | C | G | Heterozygous |
| 19 | 18897526 | A | AG | Homozygous |
| 19 | 18898675 | C | T | Homozygous |
| 19 | 18898683 | TTC | T | Homozygous |
| 19 | 41838018 | G | A | Heterozygous |
| 19 | 42863510 | G | A | Heterozygous |
| 20 | 57474722 | GCA | G | Heterozygous |
| 21 | 47845992 | T | C | Heterozygous |
| X | 49853459 | C | T | Heterozygous |

**Supplemental Table S3.** Rare variants in genes not yet linked to skeletal dysplasia and fitting autosomal recessive inheritance pattern and de novo model.

| **Gene** | **Encoded protein** | **Variant** | **Effect** | **SIFT**  **prediction** | **Polyphen-2**  **prediction** | **CADD score**  **phred** | **Inheritance**  **pattern** |
| --- | --- | --- | --- | --- | --- | --- | --- |
| *WWP1*  NM_007013.4 | WW Domain Containing E3 Ubiquitin Protein Ligase 1 | c.540-5T>C | splice-site | NA | NA | NA | AR |
| *RC3H1*  NM_172071.4 | Ring Finger And CCCH-Type Domains 1 | c.2137C>G p.(Gln713Glu) | missense | tolerated | benign | 7.6 | AR  (compound  heterozygous variant) |
|  |  | c.435A>G  p.(Val146Ala) | missense | deleterious | probably  damaging | 24.1 |  |
| *TNS1*  NM_022648.7 | Tensin 1 | c.5102C>T  p.(Thr1701Met) | missense | deleterious | benign | 34 | AR  (compound heterozygous variant) |
|  |  | c.3080A>T  p.(Glu1027Val) | missense | deleterious | possibly  damaging | 32 |  |
| *CRIPAK*  *NM_175918.3* | Cysteine-Rich PAK1 Inhibitor | c.209T>A  p.(Val70Asp) | missense | tolerated (low confidence) | benign | 0.01 | AR  (compound  heterozygous variant) |
|  |  | c.1103C>T  p.(Ala368Val) | missense | tolerated | benign | 18.75 |  |
| *SPDL1*  *NM_001329639.1* | Spindle Apparatus Coiled-Coil Protein 1 | c.59G>A  p.(Arg20Gln) | missense | tolerated | possibly damaging | 22.8 | AR  (compound  heterozygous variant) |
|  |  | c.1423G>A  p.(Gly475Arg) | missense | tolerated | benign | 1.48 |  |
| *SYT15*  NM_031912.5 | Synaptotagmin 15 | c.617G>A  p.(Ser206Phe) | missense | tolerated | possibly damaging | 22.8 | *de novo* variant |

AR=autosomal recessive; NA=not available; CADD= Combined Annotation Dependent Depletion

**Supplemental Table S4.** Rare variants (n=7) identified in skeletal dysplasia genes and fulfilling the ES filtering criteria.

| Chromosome | Position | Change | Gene | Impact | Impact severity | Polyphen | SIFT | CADD | Minor Allele Frequency | dbSNP IDs | GERP |
| --- | --- | --- | --- | --- | --- | --- | --- | --- | --- | --- | --- |
| chr2 | 15470841 | T>C | *NBAS* | Missense | MED | Possibly damaging | Deleterious | 17.21 | 0.00346202 | rs143724414 | 4.44999981 |
| chr4 | 88535073 | T>C | *DSPP* | Missense | MED | Benign |  | 0 | 0.00666926 | rs191967636 | -7.0999999 |
| chr4 | 170520286 | T>C | *NEK1* | Missense | MED | Probably damaging | Deleterious | 27.6 | 0.001 | rs201350526 | 5.90999985 |
| chr12 | 114837349 | C>A | *TBX5* | Missense | MED | Probably damaging | Deleterious | 34 | 0.0072 | rs77357563 | 4.73999977 |
| chr14 | 92470358 | A>T | *TRIP11* | Stopgain | HIGH |  |  | 35 | 4.50E-05 | rs745372938 | -1.47 |
| chr17 | 39976713 | C>T | *FKBP10* | Missense | MED | Probably damaging | Deleterious | 35 | 0.00209356 | rs146422412 | 4.88999987 |
| chr17 | 76993393 | C>T | *CANT1* | Missense | MED | Benign | Deleterious low confidence | 9.58 | 8.64E-05 | rs773657859 | -6.6199999 |

**Supplemental Table S5.** Digenic effect predictions after evaluation of eighty-seven rare variants identified in skeletal dysplasia genes in the index patient.

| **Gene A** | **Gene B** | **Classification**  **score** | **Supportscore** | **Predicted class** | **Confidence**  **zone** | **% Pat.** | **# Pat.** | **Total count** | **Median Classif.** | **Median Support** | **True digenic** | **Monogenic + Modifier** | **Dual Molecular Diagnosis** |
| --- | --- | --- | --- | --- | --- | --- | --- | --- | --- | --- | --- | --- | --- |
| *TBX5* | *TRIP11* | 0,84 | 100 | Disease-causing | 99%-zone | 100 | 1 | 1 | 0,84 | 100 | 0,911 | 0,049 | 0,04 |
| *FKBP10* | *TBX5* | 0,777390977 | 99,8 | Disease-causing | 95%-zone | 100 | 1 | 1 | 0,78 | 99,8 | 0,856 | 0,059 | 0,086 |
| *FKBP10* | *TRIP11* | 0,73 | 99,8 | Disease-causing | 95%-zone | 100 | 1 | 1 | 0,73 | 99,8 | 0,892 | 0,063 | 0,045 |
| *TBX5* | *NEK1* | 0,718652194 | 98,8 | Disease-causing | 95%-zone | 100 | 1 | 1 | 0,72 | 98,8 | 0,863 | 0,092 | 0,044 |
| *NEK1* | *TRIP11* | 0,659864865 | 96,6 | Disease-causing | 95%-zone | 100 | 1 | 1 | 0,66 | 96,6 | 0,827 | 0,104 | 0,069 |
| *FKBP10* | *NEK1* | 0,57 | 79,6 | Disease-causing | 95%-zone | 100 | 1 | 1 | 0,57 | 79,6 | 0,833 | 0,116 | 0,051 |
| *TBX5* | *NBAS* | 0,54 | 68 | Disease-causing | NA | 100 | 1 | 1 | 0,54 | 68 | NA | NA | NA |
| *TBX5* | *DSPP* | 0,43 | 28,8 | Neutral |  | 0 | 0 | 1 | 0,43 | 28,8 |  |  |  |
| *TBX5* | *GALNS* | 0,433833333 | 26,2 | Neutral |  | 0 | 0 | 1 | 0,43 | 26,2 |  |  |  |
| *FKBP10* | *NBAS* | 0,38 | 12,6 | Neutral |  | 0 | 0 | 1 | 0,38 | 12,6 |  |  |  |
| *NBAS* | *TRIP11* | 0,33 | 5,8 | Neutral |  | 0 | 0 | 1 | 0,33 | 5,8 |  |  |  |
| *FKBP10* | *GALNS* | 0,327666667 | 5,4 | Neutral |  | 0 | 0 | 1 | 0,33 | 5,4 |  |  |  |
| *TRIP11* | *DSPP* | 0,33 | 4,2 | Neutral |  | 0 | 0 | 1 | 0,33 | 4,2 |  |  |  |
| *GALNS* | *TRIP11* | 0,33 | 3,8 | Neutral |  | 0 | 0 | 1 | 0,33 | 3,8 |  |  |  |
| *NEK1* | *NBAS* | 0,283333333 | 2,2 | Neutral |  | 0 | 0 | 1 | 0,29 | 1,8 |  |  |  |
| *FKBP10* | *DSPP* | 0,291384615 | 1,8 | Neutral |  | 0 | 0 | 1 | 0,28 | 2,2 |  |  |  |
| *NEK1* | *GALNS* | 0,25375 | 1,2 | Neutral |  | 0 | 0 | 1 | 0,25 | 1,2 |  |  |  |
| *NEK1* | *DSPP* | 0,240374126 | 0 | Neutral |  | 0 | 0 | 1 | 0,24 | 0 |  |  |  |
| *NBAS* | *DSPP* | 0,02 | 0 | Neutral |  | 0 | 0 | 1 | 0,02 | 0 |  |  |  |
| *GALNS* | *DSPP* | 0,01 | 0 | Neutral |  | 0 | 0 | 1 | 0,01 | 0 |  |  |  |
| *GALNS* | *NBAS* | 0,000372294 | 0 | Neutral |  | 0 | 0 | 1 | 0 | 0 |  |  |  |

Pat.= pathogenic; Classif.= classification; NA= not available.

**Supplemental Table S6.** Biological processes associated with genes involved in skeletal dysplasia genes and pathogenic variants identified in the index patient.

| Term | Annotation | Term genes found | Input size | Term genes | Genes universe | Hypergeometric p-value | Hypergeometric adjusted p-value | Genes |
| --- | --- | --- | --- | --- | --- | --- | --- | --- |
| Cilium assembly | GO:0060271 | 42 | 379 | 205 | 18944 | 2.40E-30 | 3.48E-27 | *IFT172,TCTN3,C2CD3,RPGRIP1L,ICK,IFT140,OFD1,MKKS,C21orf2,BBS4,BBS2,BBS1,PCNT,NOTCH1,****NEK1****,TCTEX1D2,FLNA,BBS5,TTC8,IFT43,WDR34,ARL6,CEP290,TCTN2,DYNC2H1,TMEM231,WDR19,IFT80,CC2D2A,WDR35,IFT122,BBS7,WDR60,MKS1,RAB23,DYNC2LI1,TMEM216,IFT52,WDPCP,SNX10,IFT81,BBS9* |
| Bone development | GO:0060348 | 25 | 379 | 64 | 18944 | 3.02E-26 | 1.46E-23 | *IFT172,POC1A,****TRIP11****,PAPSS2,TNFSF11,WNT1,TWIST1,SPARC,RPL13,PPIB,PLS3,PHEX,LRP5,GNAS,GJA1,SH3PXD2B,ASXL1,AMER1,FGFR2,SLC10A7,P3H1,TMEM38B,DYM,ANKRD11,COL2A1* |
| Cartilage development | GO:0051216 | 27 | 379 | 80 | 18944 | 3.14E-26 | 1.30E-23 | *CHSY1,****TRIP11****,NOG,GDF5,MKKS,WNT7A,WNT5A,TGFBR2,BMPR1B,SOX9,BMP2,BMP1,BBS2,ATP7A,PITX1,MSX2,MMP13,MGP,MATN3,IHH,GNAS,TAPT1,EVC,COMP,COL11A2,COL11A1,COL2A1* |
| Cell projection organization | GO:0030030 | 35 | 379 | 193 | 18944 | 1.50E-23 | 5.43E-21 | *TCTN3,C2CD3,POC1A,ICK,IFT140,OFD1,C21orf2,BBS4,BBS2,BBS1,****NEK1****,TAPT1,FLNA,BBS5,TTC8,IFT43,ARL6,CEP290,TCTN2,DYNC2H1,TMEM231,WDR19,CC2D2A,WDR35,IFT122,BBS7,WDR60,MKS1,DYNC2LI1,TMEM216,IFT52,WDPCP,SNX10,IFT81,BBS9* |
| Intraciliary transport involved in cilium assembly | GO:0035735 | 15 | 379 | 40 | 18944 | 6.38E-16 | 1.32E-13 | *IFT172,IFT140,****TRIP11****,IFT43,WDR34,TTC21B,DYNC2H1,WDR19,IFT80,WDR35,IFT122,WDR60,DYNC2LI1,IFT52,IFT81* |
| Collagen fibril organization | GO:0030199 | 15 | 379 | 50 | 18944 | 2.98E-14 | 4.31E-12 | *CRTAP,SERPINH1,TGFB2,ATP7A,DDR2,NF1,EXT1,ACAN,****FKBP10****,COMP,COL11A2,COL11A1,COL2A1,COL1A2,COL1A1* |
| In utero embryonic development | GO:0001701 | 27 | 379 | 235 | 18944 | 2.48E-13 | 2.87E-11 | *C2CD3,RPGRIP1L,SEC24D,ACVR1,NOG,TWIST1,TGFBR2,TBX3,BMP2,PTH1R,PSPH,ATP7A,NOTCH2,NOTCH1,SMAD4,SMAD3,LMX1B,IHH,IFITM5,GLI3,FGFR2,FGFR1,TMEM231,****FKBP10****,WDR19,SALL4,ANKRD11* |
| Wound healing | GO:0042060 | 18 | 379 | 107 | 18944 | 4.04E-12 | 3.54E-10 | *CDH3,NOG,WNT7A,WNT5A,TGFBR2,TGFB2,SPARC,NOTCH2,NF1,MSX2,SMAD3,GLI3,FN1,FGFR2,FGF10,EXT1,****FKBP10****,COL1A1* |
| Protein glycosylation | GO:0006486 | 14 | 379 | 175 | 18944 | 1.20E-05 | 0.000272483 | *B3GAT3,B4GALT7,ALG3,****TRIP11****,DPM1,GALNT3,B3GLCT,B3GALT6,EXTL3,EXT2,EXT1,ALG9,ALG12,DPAGT1* |
| Chondrocyte differentiation involved in endochondral bone morphogenesis | GO:0003413 | 3 | 379 | 4 | 18944 | 3.13E-05 | 0.000595904 | ***TRIP11****,SOX9,IHH* |
| Ventricular septum development | GO:0003281 | 5 | 379 | 36 | 18944 | 0.00070635 | 0.00727209 | ***TRIP11****,WNT5A,****TBX5****,SALL1,SALL4* |
| Extracellular matrix assembly | GO:0085029 | 3 | 379 | 11 | 18944 | 0.00116325 | 0.0108208 | *TGFB1,****FKBP10****,COL1A2* |

**Supplemental Table S7.** OMIM Clinical Synopsis Table and clinical features of the index patient.

|  | **OMIM diseases** | | | | | | | | **Index patient** |
| --- | --- | --- | --- | --- | --- | --- | --- | --- | --- |
| **Phenotype MIM #** | 263520 | 613091 | 142900 | 184260 | 200600 | 259450 | 610968 | 614800 |  |
| **Disease** | short-rib thoracic dysplasia 6 with or without polydactyly; SRTD6 | short-rib thoracic dysplasia 3 with or without polydactyly; SRTD3 | holt-oram syndrome; HOS | odontochondrodysplasia; ODCD | achondrogenesis, type ia; ACG1A | bruck syndrome 1; BRKS1 | osteogenesis imperfecta, type xi; OI11 | short stature, optic nerve atrophy, and pelger-huet anomaly; SOPH | ? ODCD |
| **Gene/s** | -*NEK1* -*NEK1* +  *DYN2CH1* | -*NEK1* -*NEK1* +  *DYN2CH1* | *TBX5* | *TRIP11* | *TRIP11* | *FKBP10* | *FKBP10* | *NBAS* | *-TRIP11*  *-FKBP10*  *-TBX5*  *-NEK1*  *-NBAS* |
| **Inheritance pattern** | -Autosomal recessive - Digenic recessive | - Autosomal recessive - Digenic recessive | Autosomal dominant | Autosomal recessive | Autosomal recessive | Autosomal recessive | Autosomal recessive | Autosomal recessive | Oligogenic inheritance |
| **Height** | Dwarfism, disproportionate | Short stature (in childhood) |  | Short stature | Dwarfism, marked micromelic | Short stature | Short stature (childhood) Birth length normal | Postnatal growth failure | Short stature |
| **Trunk** |  |  |  |  | Short trunk |  |  |  |  |
| **Head** | Brachycephaly (in some patients) |  |  | Relative macrocephaly |  |  | Brachycephaly | Brachycephaly |  |
| **Face** |  |  |  | Narrow face, long philtrum, prominent forehead |  |  | Triangular face | Long face, senile- appearing face, narrow forehead, prominent glabella, hypoplastic cheekbone, small facial features, facial asymmetry, long philtrum | Mildly prominent forehead |
| **Teeth** |  |  |  | -Dentinogenes s imperfecta - Delayed tooth eruption, primary and secondary |  | Normal teeth | Dentinogenesis imperfecta (in one family) |  | Dentinogenesis imperfecta |
| **Neck** |  |  |  |  | Short neck |  |  | Short neck | Short neck |
| **External features** | - Narrow thorax - Pectus carinatum (rare) | Small thorax | Absent pectoralis major muscle | Narrow thorax | Barrel-shaped chest |  |  |  | Narrow thorax |
| **Ribs Sternum Clavicles & Scapulae** | - Short ribs - Horizontal ribs - Bifurcated ribs - Handlebar clavicles - Squared scapulae | - Shortened ribs - Horizontally oriented ribs - Handlebar clavicles | Pectus excavatum or carinatum | Pectus carinatum | -Short, fractured ribs - Beaded ribs - Short, wide clavicles - Hypoplastic scapulae  - Protuberant abdomen | Pectus carinatum |  |  | - Short ribs  - Pectus carinatum |
| **Other features** | - Markedly stunted and disorganized endochondral ossification - Hypermobile joints |  |  | - Joint hyperextensibility - Osteoporosis - Chondrocytes have large vacuoles of dilated rough endoplasmic reticulum seen on electron microscopy |  | - Osteoporosis - Bone fragility - Joint contractures, congenital (knee, ankle, hip, elbow) - Joint laxity (fingers and wrist) | - Moderate to severe bone fragility - Moderately deforming osteogenesis imperfecta - Joint laxity - Decreased bone mineral density Z score - Osteopenia | - Delayed bone age (in some patients) | - Joint laxity in the knees and fingers  - Mild tibial bowing |
| **Spine** | Platyspondyly, mild (rare) | Scoliosis | -Vertebral anomalies - Thoracic scoliosis | - Biconvex vertebral bodies - Coronal clefts (neonate) - Scoliosis - Platyspondyly (neonate) | - Unossified vertebral bodies - Cervical and upper thoracic pedicles ossified | - Kyphosis - Scoliosis - Flattened vertebral bodies - Vertebral wedging | - Wedge-shaped vertebrae - Biconcave vertebrae - Vertebral compression fractures (8/8 patients) - Scoliosis (5/8 patients) - Kyphoscoliosis |  | - Abnormal vertebrae with coronal clefts  - Kyphosis  - Progressive scoliosis |
| **Pelvis** | Underdeveloped lower pelvis (rare) | - Small ilia - Acetabular spurs |  | - Flared iliac wings - Horizontal acetabular roof - Small sciatic notch - Lacy iliac wings (early childhood) | - Pubic bones ossified - Arched ilium - Hypoplastic ischium | - Protrusio acetabuli - Coxa vara | -Unilateral/bilateral coxa vara (5/8 patients) - Protrusio acetabuli (4/8 patients) - 'Fish-scale' pattern of lamellae - Increased osteoid volume - Hyperosteoidosis |  | - Trident configuration of the pelvis, and lacy iliac wings |
| **Limbs** | - Short long bones - Mesomelic limb shortening - Disproportionate shortening of the tibia - Ovoid tibia shorter than fibula - Tibial agenesis (in some patients) - Sclerotic proximal femoral metaphyses (rare) - Metaphyseal broadening of distal femora, mild (rare) - Metaphyseal broadening of proximal tibia, mild (rare) | - Shortened long bones - Shortened femurs - Bowed femurs - Widened metaphyses - Metaphyseal spurs - Tibial agenesis - Hypoplastic ulna (in some patients) - Hypoplastic fibula (in some patients) - Ovoid tibia, shorter than the fibula (rare) | - Absent thumb - Bifid thumb - Triphalangeal thumb - Carpal bone anomalies - Upper extremity phocomelia - Radial- ulnar anomalies - Asymmetric involvement | *-* Genu vara - Genu recurvata - Short long bones - Broad, cupped metaphyses - Mesomelia - 'Banana peel' configuration of distal radius - Small, irregular epiphyses | - Micromelia - Wedged-shape femur with proximal metaphyseal spike - Short broad tibia - Short radius |  | - Bulbous metaphyses (2/8 patients) - Bowed extremities - Long bone deformity | Micromelia | - Metaphyseal flaring of all tubular bones |
| **Hands** | *-* Postaxial polydactyly - Preaxial polydactyly - Brachydactyly (rare) - Polysyndactyly | *-* Brachydactyly - Cone-shaped epiphyses - Postaxial polydactyly (in some patients) - Polysyndactyly |  | - Mild brachydactyly,  - Short metacarpals - Short phalanges - Cone-shaped epiphyses - Delayed carpal ossification | - Unossified hands |  |  | *-* Brachydactyly - Hypermobility of small joints - Simian crease (in some patients) - Syndactyly (rare) | - Brachydactyly with short and broad hands |
| **Feet** | - Hallucal and postaxial polysyndactyly - Postaxial polydactyly | - Polysyndactyly (rare) - Preaxial polydactyly (in some patients) - Postaxial polydactyly (in some patients) - Club feet (rare) |  |  | - Unossified feet | - Talipes equinovarus |  | - Brachydactyly - Hypermobility of small joints - Wide feet  - High arch  - Sandal gap  - Wide hallux | - Brachydactyly with short and broad feet |
| **Skin** |  |  |  |  |  | Pterygia (knees and elbows) | - Normal skin - No easy bruisability | - Loose skin - Decreased turgor of skin | - Redundant skin folds in the arms |
| **Molecular defects** |  | - Defect in retrograde intraflagellar transport in cilia |  | - Abnormal electrophoretic mobility of type II collagen |  |  | - Elevated serum alkaline phosphatase |  |  |
| **Severity** | - Variable features may be present - Death in perinatal period (in some patients) | - Variable severity, even within families - Thoracic abnormalities tend to improve with age - Very low occurrence of retinal, hepatic, pancreatic, and renal anomalies |  | - Possible gonadal mosaicism in one report - Mesomelia becomes more evident with age | - Infants are stillborn or die shortly after birth | - Onset of fractures in infancy to early childhood | - Onset of fractures 4-18 months of life - Severe ambulatory restriction |  |  |
